# Thoracoabdominal Approach for Large Retroperitoneal Masses: Case Series and Review

**DOI:** 10.1155/2019/8071025

**Published:** 2019-03-10

**Authors:** Siv Venkat, Andre Matteliano, Darrel Drachenberg

**Affiliations:** Section of Urology, University of Manitoba, Winnipeg, MB, Canada

## Abstract

The thoracoabdominal incision was first described in 1946 as an approach to concomitant abdominal, retroperitoneal, and thoracic injuries. In urology, this technique was popularized in 1949 for the resection of large renal tumours. Today, it is reserved for complex cases where optimal exposure of the renal hilum and adrenal and superior pole of the kidney is necessary. We present four consecutive cases in which this approach was taken by a single surgeon at our tertiary surgical centre. The outcomes, postoperative course, and pathology are described. We provide a comprehensive literature review and outline the indications, advantages, and disadvantages of this approach.* Objectives.* To present a case series outlining the efficacy and safety of the thoracoabdominal incision in complex oncologic procedures in urology.* Methods.* Four cases utilizing the thoracoabdominal incision, performed by a single surgeon at our tertiary care center, were reviewed. Case history, preoperative imaging, intraoperative experience, postoperative course, final pathology, and complications were examined. A thorough literature review was performed and comparison made with historical cohorts for estimated blood loss, length of stay, and complications encountered versus other common surgical approaches. The indications, advantages, and disadvantages of the thoracoabdominal approach were outlined.* Results.* All patients had large retroperitoneal masses of varying complexity, requiring maximal surgical exposure. Surgery was straightforward in all cases, without any significant perioperative or postoperative complications. Postoperative pain, length of hospital stay, estimated blood loss, and analgesia requirements were all similar to open and mini-flank approaches in review of historical case series cohorts. Laparoscopic approaches had lower estimated blood loss and length of stay.* Conclusions.* The thoracoabdominal approach is rarely utilized in urological surgery, due to the perceived morbidity in violating the thoracic cavity. These cases outline the benefit of the thoracoabdominal approach in select cases requiring maximal surgical exposure, and the generally benign postoperative course that appropriately selected patients may hope to endure. Postoperative pain, length of hospital stay, estimated blood loss, and analgesia requirements can be expected to be similar open and mini-flank approaches. As expected, laparoscopic approaches had lower estimated blood loss and length of stay.

## 1. Case 1

**Figure 1:**
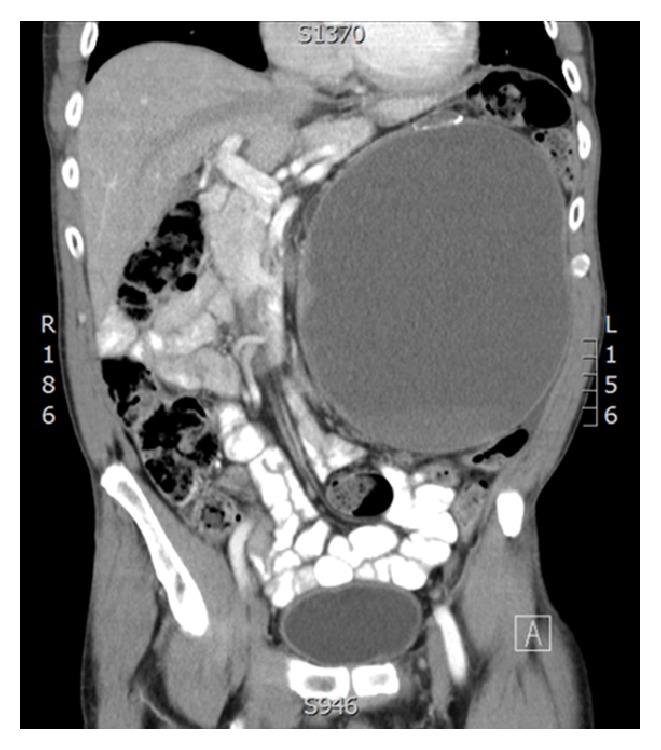
CT abdomen coronal view showing a large left cystic renal mass displacing great vessels laterally.

A 66-year-old Aboriginal male presented to his family physician with a 2-month history of early satiety, nausea, and abdominal distension. An abdominal CT scan revealed a 20 cm Bosniak IV left renal mass. This occupied much of the left hemiabdomen and displaced the great vessels laterally. No evidence of metastatic disease was found on further workup (Figure 1).

The patient underwent a radical left nephrectomy. A thoracoabdominal approach was selected due to size and superior polar location of the renal mass. No intraoperative complications were encountered, and the procedure was well tolerated. A 28 Fr chest tube was placed prior to the closure of the thoracic cavity and was connected to low suction. A nasogastric tube (NGT) was placed in anticipation of a postoperative ileus. Intraoperative estimated blood loss (EBL) was 400cc.

The patient's NGT was clamped on postoperative day 2 and removed on postoperative day 3. The epidural was discontinued on postoperative day 2, and the patient was weaned off intravenous analgesia on postoperative day 4. The following day, on postoperative day 5, the chest tube was removed. The patient was subsequently discharged on postoperative day 6 without incident for a total length of stay (LOS) of 6 days.

Final pathological analysis confirmed a type 1 papillary renal cell carcinoma. Surgical margins were negative with no evidence of lymphovascular invasion (LVI), corresponding to pathological stage T2bNxMx. Tumour grade was recorded as Fuhrman nuclear grade 2/4.

## 2. Case 2

**Figure 2:**
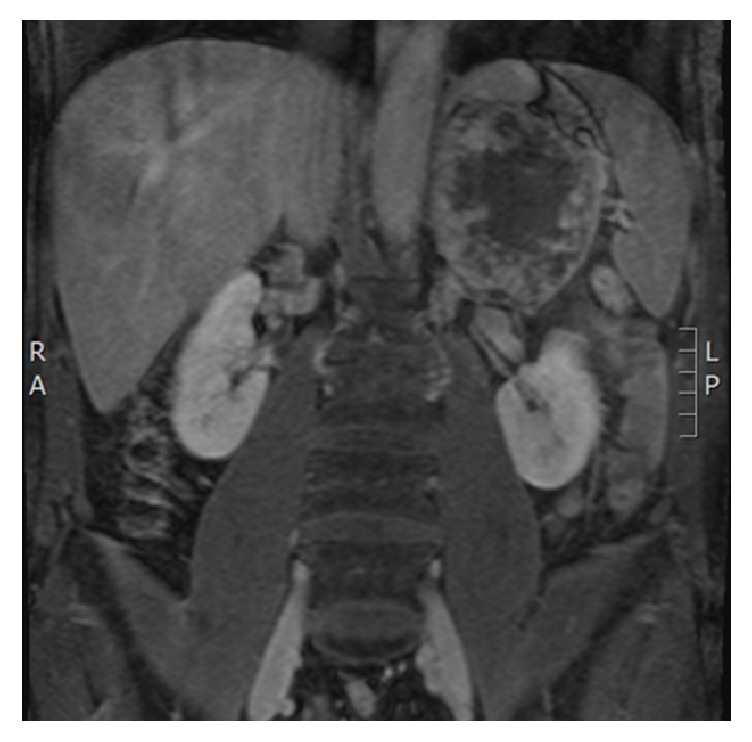
Gadolinium-enhanced T1 abdominal MRI, coronal view, showing a large heterogeneous left adrenal mass.

A 50-year-old Caucasian male with a history of hypertension and benign prostatic hypertrophy was found to have microscopic hematuria on his annual urinalysis. An abdominal MRI found an incidental 12 cm left adrenal mass involving the superior pole of the left kidney, and possibly the splenic hilum and distal pancreas. Imaging findings were concerning for a locally invasive adrenocortical carcinoma (Figure 2).

There was no evidence of lymphadenopathy or distant metastases on further workup. The patient had serum DHEAS, 17-ketosteroid, and cortisol functionality tests drawn, which were negative. Urine metanephrines were also negative, confirming a nonfunctional adrenal mass.

The patient subsequently underwent left nephroadrenalectomy. A thoracoabdominal approach was favoured due to the size, location, and locally invasive appearance of the mass. Intraoperatively, the spleen and pancreas were found to be uninvolved and did not require resection. No complications were encountered and EBL was 150cc. A 28 Fr chest tube was placed prior to the closure of the thoracic cavity and connected to low suction.

The chest tube was removed on postoperative day 3, and a follow-up radiograph confirmed the absence of a pneumothorax. The patient experienced modest difficulty weaning the epidural, which was discontinued on postoperative day 5. He was discharged on postoperative day 6 when pain was well managed with oral analgesia.

On pathological analysis, microscopic inspection revealed extensive fibrosis, hyalinization, focal dystrophic calcification, and ossification. Immunohistochemical studies (cytokeratin, S100, vimentin, and EMA) did not show evidence of neoplastic changes. Final pathological diagnosis confirmed an adrenal pseudocyst. No further follow-up was necessary.

## 3. Case 3

**Figure 3:**
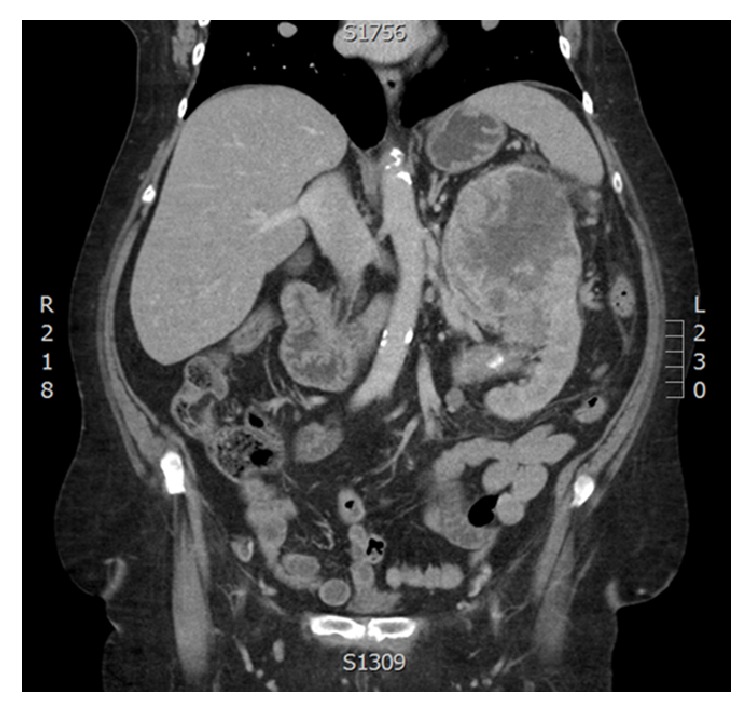
CT abdomen coronal view showing a large heterogeneous left renal mass with suspected splenic hilar invasion.

A 67-year-old Aboriginal female with a history of hypertension and diabetes presented to her family physician with a 3-month history of 20 pound weight loss, early satiety, and fatigue. A CT scan of her abdomen revealed a 14 cm mass in the superior pole of the left kidney with suspected splenic hilar invasion. There was evidence of an enhancing soft tissue mass in the tail of the pancreas, suspicious for metastasis. Further metastatic workup revealed a small burden of pulmonary disease (Figure 3).

After a thorough discussion with medical oncology and a full assessment of her functional status, the patient was enrolled in a tumour vaccine trial, which required cytoreductive nephrectomy. With the assistance of the general surgery team, she underwent a left radical nephrectomy, splenectomy, distal pancreatectomy, and retroperitoneal lymph node dissection (RPLND). A 28 Fr chest tube was placed prior to the closure of the thoracic cavity and connected to low suction. Due to the size and location of the tumour, and the suspected local invasion, a thoracoabdominal approach was pursued. No complications were encountered intraoperatively and EBL was 400cc.

The patient's postoperative course was uneventful. The epidural and chest tube were discontinued on postoperative day 4. She was weaned off intravenous analgesia by postoperative day 6 and was discharged on postoperative day 8 when fully mobile.

Final pathological analysis confirmed a clear cell renal cell carcinoma. Surgical margins were negative with no evidence of LVI. As suspected, a metastatic lesion in the distal pancreas was confirmed. Two lymph nodes were included in the analysis, both of which were negative for malignancy. Final pathological stage was defined as T3aN0M1. The tumour grade was recorded as Fuhrman nuclear grade 3/4.

## 4. Case 4

**Figure 4:**
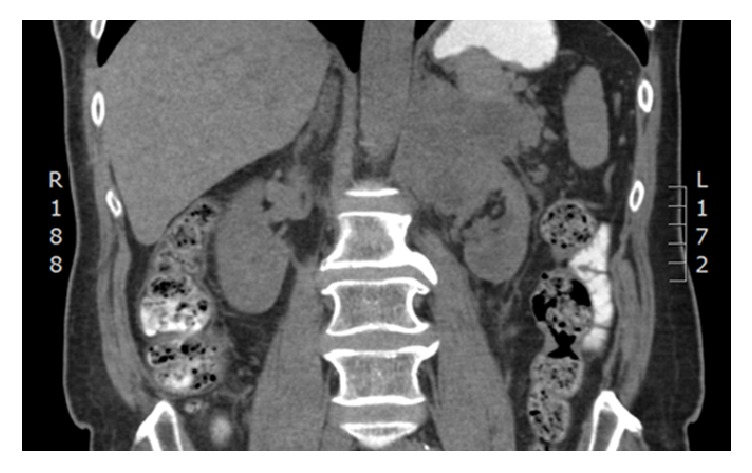
CT abdomen coronal view showing a large cystic left renal mass with suspected splenic hilar invasion.

A 61-year-old Caucasian male had previously seen a urologist for recurrent low-grade bladder cancer, which required multiple resections. Unfortunately, he was lost to follow-up and presented to his family physician several years later with abdominal discomfort and weight loss. An abdominal CT scan was ordered, which found a 10 cm cystic mass in the superior pole of the left kidney, concerning for malignancy with suspected splenic hilar invasion. A full metastatic workup was undertaken. No evidence of metastatic disease was identified (Figure 4).

The patient underwent a radical left nephrectomy, splenectomy, distal pancreatectomy, completion nephroureterectomy, and RPLND. In anticipation of a difficult resection, the thoracoabdominal approach was selected to maximize surgical exposure. Intraoperatively, the tumour was found to involve the distal pancreas, which was resected with assistance from the general surgery team. During the kidney dissection, an incidental left upper ureteric mass was identified. Given the patient's history of recurrent bladder cancer, urothelial malignancy was suspected, and a completion nephroureterectomy was performed. A 28 Fr chest tube was placed prior to the closure of the thoracic cavity and connected to low suction. No complications were encountered during the procedure and EBL was 4000cc. Three units of packed red blood cells and 1 L of fresh frozen plasma were administered intraoperatively.

The patient's postoperative course was slow, but uneventful. The epidural and chest tube were discontinued on postoperative day 5, and he was discharged on postoperative day 9, once deemed physically fit for independent living by physiotherapy and occupational therapy.

Final pathological analysis confirmed high-grade transitional cell carcinoma (TCC) with extensive tumour necrosis. Tumour was found to be invading peripelvic fat, renal parenchyma, perinephric fat, and the tail of the pancreas. The resection margins, including the pancreatic margin and the bladder cuff resection margin, were involved by TCC. Two lymph nodes were included in the specimen, which were negative for malignancy. Final pathological stage was defined as T4N0M1. The patient was referred to medical oncology for consideration of systemic therapy.

## 5. Discussion

The thoracoabdominal approach involves incision of the eighth, ninth, or tenth rib, with transpleural, transdiaphragmatic, and transabdominal exposure of retroperitoneal structures and the pleural and peritoneal cavities [1]. This method is particularly useful in right-sided cases, where hepatic veins limit exposure and control of the renal vascular supply [2]. Mobilizing the colon medially and kocherizing the duodenum allow a straightforward approach to the right kidney and early vascular control of the great vessels. For left sided renal masses, the colon and the tail of the pancreas are mobilized, providing excellent exposure of the renal pedicle [3]. This approach is advantageous in cases involving large retroperitoneal masses. In cases where surrounding structures are involved, it provides optimal exposure to allow meticulous resection [3, 4]. Specific indications for this method have been proposed, which include complex renal malignancies with inferior vena cava involvement or with local spread, a large upper pole tumour greater than 7 cm, and surgeon preference [1]. It has also been described in cases of partial nephrectomy involving upper pole masses [4].

Due to the violation of the thoracic cavity, some surgeons perceive the added morbidity of this approach as a deterrent to its use. However, there are certain advantages that this approach offers over a standard flank or transabdominal or lumbar incision. The incomparable exposure of the kidney and adrenal and renal hilum allows for early primary ligation of the renal vasculature before tumour manipulation. It also allows for easier resection of Gerota's fascia, the ipsilateral adrenal gland, and surgical extirpation of the lymphatic field [1]. Violation of the thoracic cavity also permits biopsy and resection of pulmonary lesions [2].

A commonly described disadvantage of this approach is the incision of multiple muscle layers when accessing the abdominal and thoracic cavities [1]. Inherently, this carries the risk of injuring the phrenic nerve and compromising diaphragmatic function [2, 4]. Potential injury to adjacent structures is also described. Splenic injury is possible, occurring most often during division and resection of the diaphragm. Other potential injuries include ureteric injury during retroperitoneal dissection and left first lumbar vein injury during mobilization of the left kidney. Postoperative pain is a proposed drawback of this approach, due to the transection of the cartilaginous costal arch [5].

A recent study by Yang et al. [6] compared the morbidity of various surgical incisions. In this retrospective study, the thoracoabdominal approach was compared to the flank incision for radical nephrectomy and found no significant difference in operative time, removal of surgical drains, postoperative pain scores, amount of analgesia use, length of hospital stay, and time from surgery to return to work. The only significant difference was estimated blood loss, with volumes of 150.2 cc and 209.9 cc for flank and thoracoabdominal approaches, respectively. Of note, there was a significant difference in the size of the tumours, with maximum diameters of 21.8 cm and 13.8 cm for the thoracoabdominal and flank approaches, respectively.

Kumar et al. [4] directly also compared the thoracoabdominal approach to the flank incision for radical nephrectomy through retrospective review and questionnaires. Again, there was no significant difference found in LOS, pain severity, both immediate and delayed, discontinuation of pain medication, time for patients to return to work, or complication rates. Mean length of stay was 4.0 ± 1.0 days (range 3 to 7) for the flank approach, and 4.2 ± 1.0 days (range 3 to 7) in the thoracoabdominal approach (p = 0.37), which was not statistically significant. A mix of epidural and patient controlled analgesia was used across both groups. This study failed to show a difference in morbidity of the thoracoabdominal incision as compared to the flank incision, but did recommend further prospective studies to be performed. This study also demonstrated compelling results for* not* placing a chest tube in patients unless there was evidence of a parietal pleural injury. In this study, patients without chest tubes had a significantly shorter postoperative recovery course, despite an increased incidence of asymptomatic pneumothoraces and pleural effusions.

Our four cases presented had EBL values of 400, 150, 400, and 4000cc, respectively, for a mean of 1237 ± 1598cc (Table 1). The large variance in this small series is due to the 4000cc blood loss in the rather complex Case 4, described above. Other studies have also looked at estimated blood loss with various approaches. Wang et al. [7] retrospectively reviewed 194 patients who underwent partial nephrectomy, comparing a mini-flank supra-12^th^ rib approach to traditional open and laparoscopic approaches. Average EBL was 103 ± 88cc for the mini-flank, 226 ± 264cc for open, and 119 ± 150cc for laparoscopic approaches. DiBlasio et al. [8] retrospectively reviewed a mini-flank supra-11^th^ rib incision for open partial and radical nephrectomy. Average EBL was 400cc. Stifleman et al. [9] reviewed a series comparing hand-assisted laparoscopic donor nephrectomy to an open flank approach. Average EBL was 83 ± 62cc for the hand-assisted laparoscopic approach and 364 ± 449cc for the flank approach. Parra et al. [10] retrospectively compared open flank and laparoscopic approaches for radical nephrectomy. Average EBL was 140cc for the laparoscopic and 295cc for the flank approach. These compare favorably with our series of thoracoabdominal cases, excluding Case 4 as described previously. As expected, the laparoscopic series did have a lower EBL. Given the small size of our series, more robust statistical analysis did not reach significance.

Our four cases had LOS values of 6, 6, 8, and 9 days, for a mean of 7.2 ± 1.3 days (Table 1). In considering postoperative length of stay, Wang et al. [7] had a mean LOS of 6.8 ± 2.1 days for mini-open approaches, 8.2 ± 3.9 days for open approaches, and 6.7 ± 2.5 days for laparoscopic approaches. DiBlasio et al. [8] had a median LOS of 5 days for their mini-flank supra 11^th^ rib incision. Stifleman et al. [9] had an average LOS of 3.5 ± 0.7 days for the hand-assisted laparoscopic approach and 4.5 ± 1.2 days for their open flank approach. Parra et al. [10] had an average LOS of 3.5 days for laparoscopic and 8 days for their open flank approach. These values are similar to our series of thoracoabdominal cases. Again, as expected, the laparoscopic series had a shorter LOS.

The four cases presented here had no complications, early or late (Table 1). Yang et al. [6] had a complication rate of 0% in the flank group and 3% in the thoracoabdominal group, with all being unrelated to the incision. Kumar et al. [4] had a complication rate of 0% in the flank group and 7% in the thoracoabdominal group, with all being unrelated to the incision. Wang et al. [7] found a complication rate of 12% for the mini-flank supra-12^th^ approach, 13% for the open, and 21% for laparoscopic, with all complications being Clavien I-III. DiBlasio et al. [8] had no intraoperative complications, with a 4% rate of late complications, all related to surgical site hernias. Stifleman et al. [9] had a total complication rate of 13% for the open group, and a total complication rate of 8% for the laparoscopic group, all Clavien II or III, with one intraoperative complication in the open group and the rest all postoperative. Parra et al. [10] had a complication rate of 16% for the laparoscopic approach, with all of these being Clavien III, and a rate of 15% for the open flank approach, with all of these being Clavien II. These series again compare favorably to our small series of thoracoabdominal cases.

## 6. Conclusion

These case reports represent clinical scenarios where the thoracoabdominal surgical approach was indicated. All patients had large retroperitoneal masses of varying complexity, requiring maximal surgical exposure. The thoracoabdominal approach is rarely utilized in urological surgery, due to the perceived morbidity in violating the thoracic cavity. However, comparison with several retrospective series examining open and mini-flank approaches suggests no difference between thoracoabdominal and the open flank approaches in terms of postoperative pain, length of hospital stay, EBL, analgesia requirements, return to work, and complication rates. As expected, laparoscopic approaches had lower EBL and LOS. Although a small series, our cases outline the benefit of the thoracoabdominal approach in select cases, and the generally benign postoperative course that appropriately selected patients may hope to endure. In the era of robotic assisted and minimally invasive surgery, urologists should be reminded of this effective and safe approach to address challenging retroperitoneal masses.

## Figures and Tables

**Table 1:** Comparison of case series and surgical approaches.

*Series*	Approach used	Number of patients	EBL (range), cc	LOS (range), days	Clavien I-III complications, %
*Venkat et al., 2018*	Thoracoabdominal	4	1237 ± 1598 (150-4000)	7.3 ± 1.3 (6-9)	0
*Yang et al., 2009 *	Thoracoabdominal	60	150 ± 10	9.5 ± 1.6	3
*Kumar et al., 1999*	Thoracoabdominal	42	-	4.2 ± 1.0 (3-7)	7
*Yang et al., 2009 *	Open Flank	56	209 ± 13	9.4 ± 1.6	0
*Kumar et al., 1999*	Open Flank	52	-	4.0 ± 1.0 (3-7)	0
*Wang et al., 2014*	Open Flank	111	226 ± 265 (10-3000)	8.2 ± 3.9 (3-39)	13
*Stifleman et al., 2001*	Open Flank	23	364 ± 449	4.5 ± 1.2	13
*Parra et al., 1995*	Open Flank	13	295 (75-750)	8.0 (3-16)	15
*Wang et al., 2014*	Mini-flank, supra 12^th^ rib	41	103 ± 88 (20-500)	6.8 ± 2.1 (5-17)	12
*DiBlasio et al., 2005*	Mini-flank, supra 11^th^ rib	167	400 (50-2400)	5.0 (3-28)	4
*Wang et al., 2014*	Laparoscopic	42	119 ± 150 (20-800)	6.65 ± 2.5 (3-16)	21
*Stifleman et al., 2001*	Laparoscopic, hand assisted	40	83 ± 62	3.5 ± 0.7	8
*Parra et al., 1995*	Laparoscopic	12	141 (50-200)	3.5 (2-6)	16
